# Flexible Flatfoot—Epidemiology, Pathophysiology and Clinical Aspects: A Review Focused on Less-Invasive Surgical Treatment in Children

**DOI:** 10.3390/children13070881

**Published:** 2026-06-30

**Authors:** Fernando De Maio, Enrico Micciulli, Davide Lardo, Ernesto Ippolito

**Affiliations:** 1 Deparment of Orthopaedic Surgery, University of Rome “Tor Vergata”, 00133 Rome, Italy; demaio@med.uniroma2.it; 2 Pediatric Hospital Bambino Gesù, Rome-Palidoro, Fiumicino, 00050 Rome, Italy; 3 Military Hospital of Rome “Attilio Friggeri”, 00184 Rome, Italy

**Keywords:** flexible flatfoot (FF), FF epidemiology, FF etiopathogenesis, FF physiopathology, FF clinical aspects, FF treatment, FF subtalar arthroereisis

## Abstract

**Highlights:**

**What are the main findings?**

- Most pediatric flexible flatfeet are asymptomatic and undergo spontaneous longitudinal arch development during the first decade of life, with a low prevalence of truly symptomatic cases.
- A discrepancy seems to exist between epidemiological data indicating few symptomatic cases and the high number of children undergoing less-invasive surgical procedures for flexible flatfoot.

**What are the implications of the main findings?**

- Indications for surgical treatment of pediatric flexible flatfoot should be restricted to cases with persistent symptoms after accurate differential diagnosis and failed conservative treatment.
- Increased awareness among physicians and families is required to prevent overtreatment in a largely benign developmental condition like flexible flatfoot that occurs mainly in children under 9–10 years of age.

**Abstract:**

Background/Objectives: To give the proper context to children’s flexible flatfoot (FF), considered to be a crippling deformity by some authors and a variation of normal foot development by others, it requires treatment only in a few symptomatic cases. During the last 20 years, according to the literature, less-invasive surgery (LIS) has been extensively performed on FF to restore the longitudinal arch of the foot, to improve FF’s function, and to prevent its presumed unfavorable evolution into adulthood. Methods: The literature was searched using the following keywords and phrases: FF, FF epidemiology, FF etiopathogenesis, FF physiopathology, FF diagnosis, FF treatment, and FF subtalar arthroereisis. The primary sources were PubMed and The Cochrane Library. Results: Epidemiological studies carried out on many people, some of them performed in a military environment, reported FF prevalence in adults of about 15%, but most flexible flatfeet (FFT) were found to be asymptomatic. In the pediatric population, FF prevalence is higher, reaching almost 40–45% up to 6 years of age and decreasing thereafter with increasing age, owing to the spontaneous formation of the longitudinal arch of the foot. Like adults, children and adolescents with FF are rarely symptomatic. These data seem to be in contrast with the high number of symptomatic children and adolescents with FF who are treated by LIS. However, epidemiological studies reflect general populations while less invasive surgical series represent selected referral cohorts. Conclusions: According to the literature, several studies on LIS report high numbers of FF operated on every month. These data raise concerns; therefore, we believe that indication for LIS must be carefully evaluated in the future to avoid overtreatment of FF.

## 1. Introduction

During the last 20 years, flexible flatfoot (FF) has been increasingly treated by less-invasive surgery (LIS) in children and adolescents [1]. Currently, a similar trend has been observed in the second half of the past century where overtreatment was mainly conservative, consisting of insoles and orthopedic shoes [2], whereas today, flatfeet (FFT) appear to be overtreated by LIS [3]. The latter is based on exosinotarsal screws and endosinotarsal implants, which are an evolution of Grice subtalar arthroereisis (SA) [4]. These surgical techniques aim to rebuild the longitudinal arch of the foot by fixing both the medio-plantar shift of the talus and the calcaneal valgus, but, at the same time, limit the movement of the subtalar joint [4].

Both parents and physicians are concerned about FF, owing to the belief that it is a foot deformity that causes functional problems in children and adolescents [5,6], although that belief is not substantiated in the literature [7,8,9].

Another important cause for concern is that FF may worsen in adulthood, overburdening parents with the responsibility of preventing such an evolution by choosing appropriate treatment during childhood [10,11].

In this article, we attempt to provide proper context for FF, which, in our opinion and according to other reliable authors [12,13,14], should not be considered to be a true foot deformity, but rather a variation of the foot’s anatomy that sometimes becomes symptomatic, thus requiring treatment. To give the correct indication for the treatment of FF by LIS, owing to the increase in its frequency in recent years [1], we reviewed the literature on the epidemiology, etiopathogenesis, pathophysiology, clinical aspects, and treatment of this condition. The FF cases illustrated in this review are taken from the authors’ archives.

## 2. Materials and Methods

As this is a narrative review with a critical appraisal on less-invasive surgery, PRISMA methodology was not followed. Regarding the research method and sources of information, three authors of this review (F.D.M., E.M. and D.L.) searched the literature by consulting the Medline (PubMed), Cochrane Library and Google Scholar databases. Pertinent pamphlets retrieved from the websites of large hospitals and universities and foot and ankle textbooks were also considered and are quoted in the References section. This research was done independently by each of the three authors from 1 January 1952 to 1 November 2025.

To build an effective search string, the following keywords and phrases were used in various combinations to obtain the most pertinent articles: flexible flatfoot (FF), FF epidemiology, FF etiopathogenesis, FF pathophysiology, FF diagnosis, FF conservative treatment, FF less-invasive surgery, and subtalar arthroereisis. No language restrictions or filters were applied. The search included epidemiological studies carried out on large numbers of individuals and etiopathogenetic, pathophysiological, clinical and conservative treatment studies, systematic reviews, and meta-analyses, privileging those with a level of evidence of II–III and that attempted to minimize the risk of bias. Clinical studies treating syndromic FF and those with differential diagnosis from other pathologic and painful coexisting foot conditions mimicking symptomatic FF were also taken into consideration.

The search was particularly focused on less-invasive surgical treatment of FF by subtalar arthroereisis, mainly including studies published from 2006 to 2025, regardless of their level of evidence, which was most frequently level IV. A small number of articles on the surgical treatment of FF by the Evans-Mosca procedure and triple arthrodesis were also reviewed.

The three reviewers retrieved the data and independently analyzed each study selected; instances of disagreement were resolved by discussion among the four authors. When no data could be found on the peculiar aspects of the topic by the academic search engines, the authors’ opinion was called upon to fill the gap, but it was labeled as expert opinion (level of evidence V) and was not included in the evidence-based conclusions.

Response letters, comments, case reports and other articles dealing with surgical treatment of FF other than those previously mentioned, as well as articles dealing with the treatment of pathologic FF, were not considered. Institutional Review Board approval was obtained before starting this review, and written informed consent was obtained either from patients or their tutors for the publication of anonymized images.

## 3. Results

### 3.1. Epidemiology

In 1947, Harris and Beath published a historical study on 3619 recruits of the Canadian Army. They reported an FF prevalence of 14.5% [15], with 91% of mild asymptomatic forms and only 9% of severe symptomatic forms. More recently, Lakstein et al. [16] published a similar study on 97,279 recruits in the Israeli Army. The authors confirmed Harris and Beath’s data with a FF prevalence of 15.3%. They also reported 95% of mild or moderate FFT and only 5% of severe forms.

Staheli et al. showed that the plantar longitudinal arch forms spontaneously during the first decade of life [17]—data confirmed by Vanderwilde et al. in a radiographic study [18]. All of the children examined by both authors were asymptomatic. In Ethiopia, Birhanu et al. found an FF prevalence of 10.27% in 1022 adolescents whose ages ranged from 11 to 18 years, and only a few FFT cases were symptomatic [19]. In China, in a transversal study carried out on 1059 children and adolescents whose age ranged from 6 to 13 years, Yin et al. found that FF prevalence decreased spontaneously from 39.5% at the age of 6 years to 11.8% at the age of 12 years [20]. In India, Aenumulapalli et al. found an FF prevalence of 13.6% in 500 adults; all of the FFT examined were asymptomatic [21].

### 3.2. Pathoanatomy

According to various authors, the classic definition of FF is a decrease in the height of the plantar longitudinal arch of the foot formed by the talus, the tarsal navicular, the cuneiforms, and the first three metatarsals, with the static support of the joints’ capsules and ligaments of the medial aspect of the tibio-tarsal and the Chopart joints, as well as of the plantar fascia [12,13,14,22,23]. Classic electromyographic studies have shown that none of the extrinsic and intrinsic muscles of the foot exert any static support, but they rather become dynamically involved only during walking [24,25].

The anatomic structures responsible for the decreased height of the longitudinal arch are as follows: (1) The deltoid ligament. (2) The calcaneo-navicular ligament or spring ligament, formed by an outer fibrous layer and an inner fibrocartilaginous layer. This ligament, together with the posterior articular surface of the navicular, forms a concave slot for the talar head called “coxa pedis”, in analogy with the hip joint. (3) The articular capsules of the ankle, subtalar, and talonavicular joints. (4) The plantar fascia [23,26]. The laxity of all the above-mentioned fibrous and fibrocartilaginous structures is responsible for both the medial and plantar shift of the talus and the deviation in valgus of the calcaneus [23,26].

In FF, ligament laxity can be either specifically localized to the foot and ankle or part of a generalized increased laxity of multiple joints, often familial [27] and associated with obesity [28]. Being overweight increases the risk of developing FF from twofold to fourfold; overweight individuals put more pressure on the longitudinal arch of their foot, overstretching the weak FF ligaments [28].

Severe FFT cases are associated either with benign congenital hypotonia [29] or with diseases of the connective tissue matrix [30,31,32], while Nourbakhsh et al. [33] showed an association between FF and rotational changes of the lower limb, often causing inward rotation of the hip, femur, and tibia with external rotation of the foot, producing an out-toeing gait.

### 3.3. Pathophysiology

Both capsules and ligaments of the tibio-tarsal and tarsal joints are stretched on the medial side of the hindfoot, becoming longer than normal. According to Huson’s law [34], if the range of motion of one tarsal joint is increased, the overall tarsal ROM will also increase, as in FF. Consequently, both the talus and the calcaneus become unstable. Under the body’s weight, the talus shifts medially and downwards at the level of the “coxa pedis” due to the subsidence of the spring ligament, while the calcaneus, made unstable by the laxity of both the deltoid and the interosseous talo-calcaneal ligaments, rotates in valgus in the subtalar joint [35]. While walking, in the stance phase, the hindfoot rotates inwards, owing to both the medial and downward shift of the talus and the valgus position of the calcaneus, while the forefoot, pushed in supination and in external rotation, must pronate to bearing weight on the metatarsal heads. The consequence of this is out-toes-walking, typical of people with FF [35].

In FFT with long-standing calcaneus valgus, foot dorsiflexion is limited by contracture of the triceps surae and tightening of the Achilles tendon. In those cases, with the calcaneus in a neutral position and the knee extended, FF drops into equinus (Silfverskiöld sign) [15].

### 3.4. Clinical Aspects

#### 3.4.1. Diagnosis

In a bipedal standing position, the longitudinal arch of the foot is either lowered or absent, with a deviation in valgus of the calcaneus. In the same position, Jack’s test is performed by passive dorsiflexion of the big toe. In FF, this maneuver elevates the longitudinal arch [22]. With the patient lying down in a prone position, the foot is manipulated to assess the typical FF flexibility, attempting passive reconstitution of its longitudinal arch, which may also be actively reconstituted by asking the patient to walk on tiptoes [22]. To further assess foot motion and muscle activity, the patient is asked to walk on their heels, to jump on one foot at a time, and to run. In asymptomatic FF, all of these tests may be easily accomplished and painless [22].

#### 3.4.2. Instrumental Assessment

A footprint, made after dipping the foot in a tray filled with dye, is the simplest method to confirm FF clinical diagnosis [15]. By using this method, FF may be classified into one of four grades of increasing severity [36] or graded using a plantar arch index [17]. Pedobarometry is a more sophisticated dynamic technique that is able to detect high-load plantar points by means of loading sensors on a treadmill on which the patient is asked to walk [37]. Radiology allows for a more precise FF definition [22]. Lateral and dorsoplantar standing views of the foot can identify the angles that define the foot’s architecture. Meary’s and pitch angles on the lateral view and talonavicular coverage angle on the dorsoplantar view, respectively, measure less than 0°, less than 15°, and more than 30° in FF [22]. Radiographs of the foot on tiptoes is an accurate investigation to assess the dynamic restoration of normal angles in FF [38]. Weight-bearing 3D CT scans are performed using cone beam technology (CBCT) with precise evaluation of hindfoot alignment angles in severely deformed FFT [39]. Kothari et al. [40] studied FF morphology by MRI, showing that, in one third of 84 children with FF, the anterior facet of the subtalar joint was missing.

#### 3.4.3. Pain

Symptomatic FF is characterized by pain that is usually triggered by long-distance walking, running, or sport activities [41]. Overstretching of both the tibialis posterior tendon and medial hindfoot ligaments, overpressure on the medial part of the calcaneal tuberosity, and overpressure on the lateral aspect of the subtalar joint are all possible causes of pain [41]. At the physical examination, all of the above-mentioned anatomical structures are tender under finger pressure, and local pain is triggered by endurance tests like jumping on the FF [41,42]. Symptomatic flatfoot mainly refers to the rigid pathologic form caused by tarsal coalition. Both radiographic and CT scan investigations must rule out pathological flatfoot [12].

#### 3.4.4. Fatigue

Fatigue could be caused by flattening of the longitudinal arch of the foot, which in turn causes a decrease in the ground reaction force [43]. The compensatory increased activity of some activator muscles of the foot can produce fatigue during walking [43]. Murley et al. [44], in an electromyographic study of two groups of adults aged 18–47 years, one with FF and the other with a normal longitudinal arch, showed that, in FF during gait, the contractile activity of the tibialis posterior increases while that of the peroneus longus decreases. The opposite happens in people with a normal longitudinal arch, while the contractile activity of the tibialis anterior does not show any significant difference between the two groups. Consequently, overall muscle energy consumption does not show a significant increase in FF [44]. Furthermore, Kirmizi et al. [45] observed, using gait analysis, pedobarographic differences after strenuous exercise of the calf muscles between FFT and normal-arched feet. They concluded that plantar pressure variables are affected differently by fatigue in the two groups, without showing overloading in FFT.

#### 3.4.5. Differential Diagnosis

**Figure 1:**
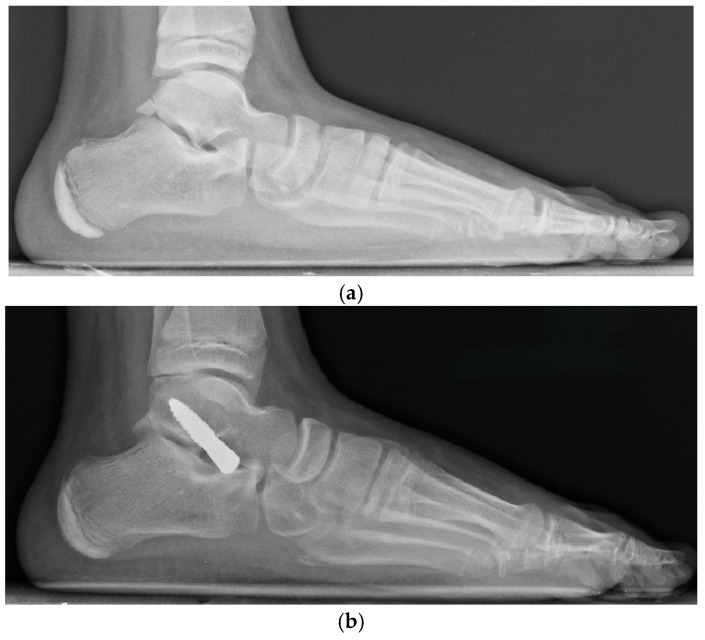
(**a**) Standing lateral radiograph of the left foot of an 11-year-old boy complaining of pain on the posterior aspect of the calcaneus. His diagnosis was symptomatic flatfoot. Meary’s and pitch angles measured, respectively, −4° and 14°. (**b**) The boy underwent surgery with an exosinotarsal talar screw, but symptoms persisted 8 months later when first seen by us. The radiolucent area present around the screw did not cause any further discomfort to the boy because tenderness was localized at the insertion of the Achilles tendon on the calcaneus. The patient was affected by Sever’s disease with a mild asymptomatic flatfoot.

**Figure 2:**
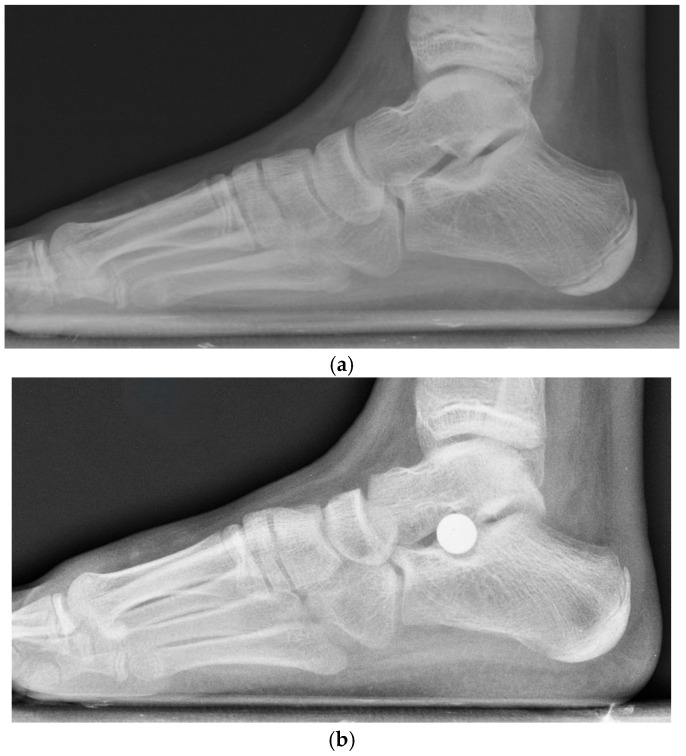
(**a**) Standing lateral radiograph of the right foot of a 12-year-old girl with a clinical history of recurrent ankle sprain and occasional pain on the lateral aspect of the ankle. She had a misdiagnosis of flatfoot because Meary’s angle measured −4°, while the other angles indicating flatfoot were normal. (**b**) An endosinotarsal implant was applied, with overcorrection of the misdiagnosed FF. As a result, ankle sprains became more frequent, and ankle pain worsened.

FF must be differentiated from rigid flatfoot caused by congenital tarsal coalitions, also defined as pathological flatfoot [12]. The latter is caused by either fibrocartilaginous, cartilaginous, or bony connections between the tarsal bones that develop during the embryonic stage. Pathological flatfoot is characterized by pain and stiffness, and it rarely becomes symptomatic before 9–10 years of age [12]. Clinical suspicion must be confirmed by MRI showing tarsal coalitions. Its prevalence ranges from 5% to 10% of all flatfeet [12,46]. Peroneal spastic flatfoot is a rigid form of symptomatic FF that may clinically mimic pathological flatfoot, but, in this case, MRI did not show tarsal coalitions [47]. External rotation of the leg bones causes out-toeing, just like FF. A physical examination is sufficient to differentiate the two conditions [48]. Between 3 and 6 years of age, pain at the level of the tarsal navicular may be caused by Kohler I disease. In cases of tenderness on the tarsal navicular, x-rays of the FF must be done to differentiate the two conditions [49]. At the same level, mostly after 9–10 years of age, pain may be caused by an accessory navicular, which often becomes symptomatic following a sprain. In this case, the bony bump appearing at the level of the navicular is not the prominence of the longitudinal arch of the foot, but rather the accessory navicular itself; either x-rays or a CT-scan must be done for a differential diagnosis [49]. Between 8 and 13 years of age, a traction apophysitis of the accessory ossification nucleus of calcaneus, or Sever’s disease, may be associated with FF. Patients report pain in the posterior aspect of the calcaneal tuberosity, which is tender at the insertion of the Achilles tendon ([49], Figure 1). In the same area, os trigonum syndrome might be differentiated. Os trigonum is an accessory ossicle of the ankle originating from a failed fusion of the secondary center of ossification of the posterolateral talar tubercle. By palpation, postero-lateral tenderness is present on the calcaneus, and forced plantar flexion of the ankle triggers pain. Radiographs of the ankle clarify diagnosis [49]. In both adolescents and adults, especially if practicing sports activities, a plantar fasciitis may mimic painful FF. Either sonography or MRI are needed for differential diagnosis [50]. Growing pains in children with FF must also be differentiated from symptomatic FF. They affect children ranging in age from 3 to 12 years. Symmetrical pain is typically reported as being located in the lower limbs mainly in the afternoon, evening, and even during the night. None of the tests previously described for physical examination of FF will be painful in children with growing pain [47]. Freiberg disease must also be differentiated; metatarsalgia is its main symptom. Tenderness is mainly located at the second metatarso-phalangeal joint and x-rays, which only become positive a few weeks after the onset of symptoms, show increased radiodensity and fragmentation of the secondary ossification center of the second metatarsal [49]. Among painful pathologies, osteochondral lesions of the talus, mostly arising around 9–10 years of age, must be excluded, as well as osteoid osteoma, which may affect foot bones during childhood and adolescence [51]. Chronic ankle sprain must also be differentiated (Figure 2).

Pain may sometimes be reported as a symptom by young overweight people who lead a sedentary life and avoid sports by complaining of FF pain. An opposite condition may also occur, i.e., non-competitive younger patients, influenced by the media or by a friend with FF, who believe that FF treatment may improve their performance in sports. Treatment may also be requested for cosmetic reasons, mainly by girls.

In cases of patients with FF who complain of easy fatigability after walking or running, a coexistent neuromuscular disease should be suspected and investigated [52].

### 3.5. Treatment

#### 3.5.1. Conservative Treatment

Staheli and Giffin [53] and Wenger et al. [2] showed that conservative treatment of children with FF by the infamous “rigid custom-made corrective orthopedic shoes” was useless because “orthopedic shoes” were lacking any corrective power [2,53]. Afterwards, Driano et al. [54] also showed that treatment with “orthopedic shoes” caused psychological problems [54].

In a systematic review of 2023, Molina-García et al. [55] concluded that there was evidence for the efficacy of foot orthoses in commercial shoes as a treatment for pediatric FFT signs and symptoms, but there was no ideal type of foot orthosis, although all had in common the incorporation of a large internal longitudinal arch support. In 2023, Oerlemans et al. [56] carried out another systematic review and meta-analysis on the effect of foot orthoses on FF in children and adults, including only randomized controlled trials and prospective studies. They concluded that, due to the heterogeneity of study designs, it was not possible to state whether foot orthoses are useful for FF in both children and adults; they may only decrease pain in adults. In a 2024 meta-analysis, Hu et al. [57] concluded that orthopedic insoles showed good efficacy for treating school-age children with symptomatic FF, but insoles custom-made using 3D-printed technology exhibited better results than prefabricated insoles, mainly in children with high BMI. Similar results were reported by Xu et al. [58] in adults. In conclusion, foot orthoses provide comfort, improve function, and can reduce pain. Adults and adolescents FFT need robust or specific custom-made orthoses, while younger children only need standard inserts. Significant pain relief may be obtained most frequently in adults. No orthotic treatment at any age can permanently fix the flatness of the longitudinal arch of the foot, although orthoses provide a type of treatment that often satisfies the parents of young patients.

In a recent systematic review of the literature on physical therapy consisting of exercises aiming to strengthen both the extrinsic and intrinsic foot muscles, Molina-García et al. [59] included 11 randomized controlled trials with a sample of 419 children aged from 6 to 14 years with FF. They concluded that functional re-education represents an effective treatment option for FF by improving both symptoms and foot functionality. Stretching exercises are also indicated in cases of tight Achilles tendon [13].

In peroneal spastic FF, after excluding tarsal coalitions and other pathologies, the recommended conservative treatment [60] is foot manipulation under general anesthesia, associated with the injection of both local anesthetic and long-acting steroids into the sinus tarsi, followed by a molded plaster cast immobilization for 4–5 weeks.

#### 3.5.2. Surgical Treatment

Two systematic reviews and meta-analysis published in 2024 reported most of the articles on LIS in FF in children [3,61]. Non-reported articles published in 2024, as well as articles published in 2025, were added by searching the search engines [62,63,64,65,66,67,68,69,70,71,72,73,74,75,76,77,78,79,80,81,82,83,84,85,86,87,88]. In almost all of the studies, children and adolescents with symptomatic FF were included. Their average age at surgery ranged from 9.5 to 13.7 years, but children ranging in age from 5 to 8 years were also included [62,63,64,65,66,67,68,69,70,72,74,75,76,77,79,81,82,84,86,87,88]. In some studies, the number of FFT cases operated on ranged from 2 to 13 cases every month [66,67,71,74,81,82,83,85]. Other inclusion criteria for surgery, regardless of the presence of symptoms, were altered radiographic angles indicating FF [64,67,88], Staheli index > 1 [67], and abnormal pedobarography indicating FF [79]. A summary of the most representative LIS studies is shown in Table 1. We were able to find only one recent study on LIS carried out in North America by Sullivan et al. in 2024 [89]. The study reports on 37 symptomatic FFT cases operated on during a 5-year period, and the youngest patient was 9 years old.

In severe long-standing FFT in which both clinical and radiographic dynamic tests do not show correctability of the longitudinal arch flatness, surgical techniques more complex than SA may be required, such as the Evans–Mosca procedure [90], while triple arthrodesis is the most recommended surgical technique in adults [91].

Surgical treatment is also indicated in peroneal spastic FF in cases of recurrence after conservative treatment [60].

Complications around LIS include persistent sinus tarsi pain, peroneal spasm, implant malposition, overcorrection, undercorrection, wrong implant size, loss of implant position, screw breakage, and, for bioresorbable screws and implants, fracture, debris from wear, loosening, foreign body reaction, arthritis, and synovitis. The prevalence of these complications ranges from 0.07% to 29.9% (Table 1).

## 4. Discussion

**Figure 3:**
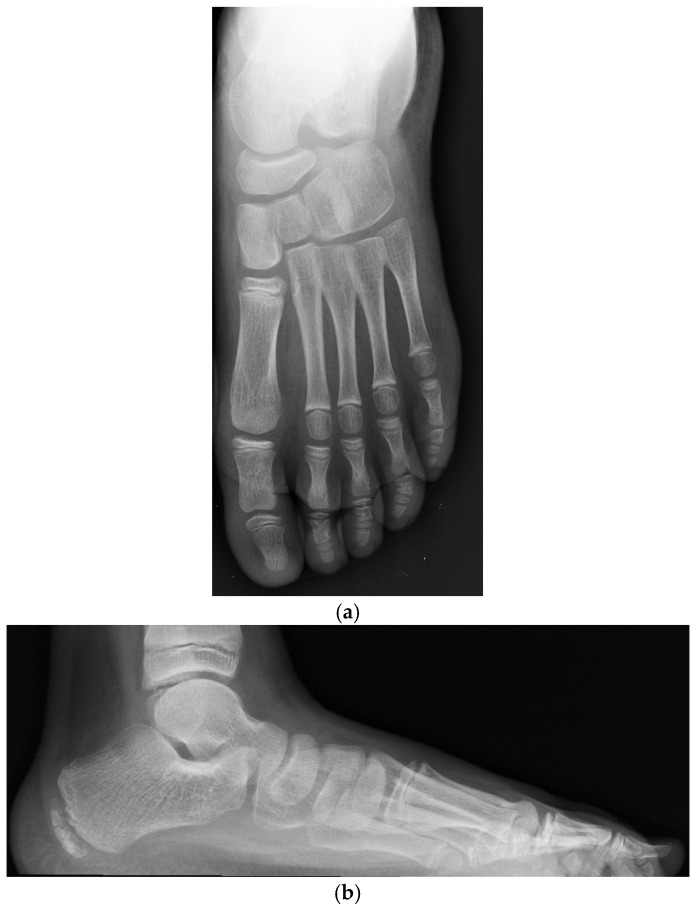

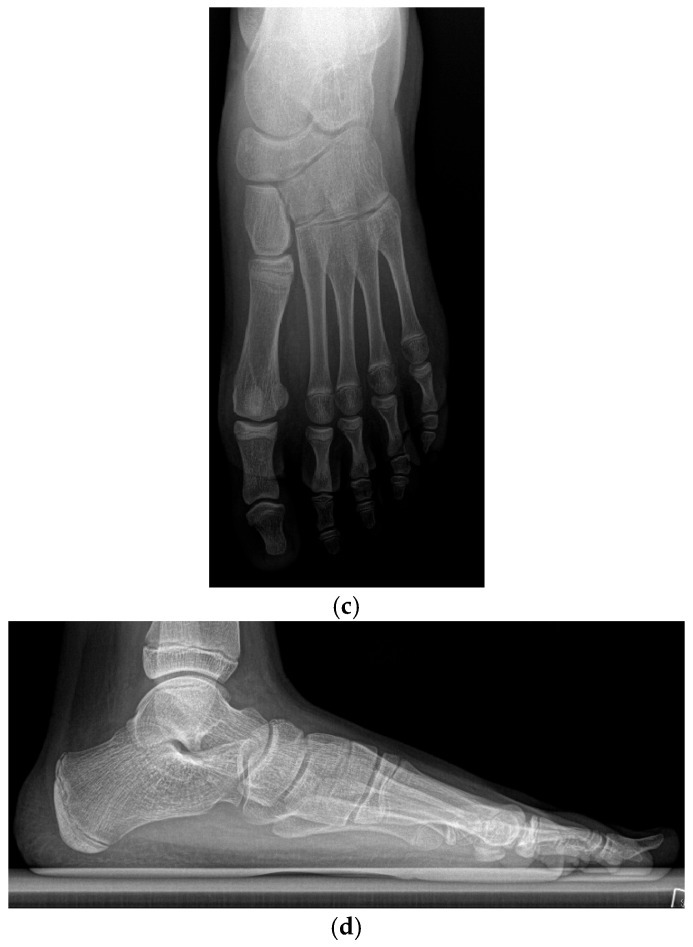
(**a**,**b**) Antero-posterior and lateral standing radiographs of the left asymptomatic flatfoot of a 7-year-old boy. The talo-navicular coverage angle measured 42°, while Meary’s and pitch angles measured, respectively, −14° and 13°. Less-invasive surgery was planned, but it was never performed. (**c**,**d**) The same foot when the boy was 14 years old. The medial arch of the foot self-recovered without any treatment, as shown by the talo-navicular coverage angle, Meary’s and pitch angles that measured respectively 29°, 0° and 20°.

Epidemiological studies, carried out all over the world, have highlighted three very important points: (1) In children, FF prevalence decreases spontaneously during the first decade of life because the longitudinal arch of the foot forms by itself with growth in that period [17,18,20] (Figure 3). (2) In adults, FF prevalence varies from about 12% to 15% of the general population [15,16,21]. (3) Most FFT are asymptomatic, with a low prevalence of symptomatic cases [15,16,17,18,19,21]. The studies by Harris and Beath and Lakstein et al. [15,16] have been disapproved by Giannini and Ceccarelli [92], who made the following remarks: “The assessments usually were based… on selected individuals such as soldiers, and therefore the assessments were about functionally good feet”. “In some cases patients become used to their relative disability and change their habits…”. “Feet with excessive pronation can be painful or, if not painful, may very likely lead to future problems, and thus should be treated” [92]. However, these remarks have limited evidence because both studies [15,16] were carried out on recruits before their enrollment as soldiers while, as far as we know, there is no evidence that children will systematically have problems in adulthood with FFT or with any other pathology of the locomotory apparatus caused by FF.

FF etiopathogenesis is still unknown. In fact, no histologic, histochemical, or biochemical study has been done so far to explain why, in FF, the fibrous structures supporting the longitudinal arch of the foot are lax. FF is often associated with the generalized joint laxity, which is a familial condition characterized by joint hypermobility, the pathogenesis of which is also unknown [27]. In cases of generalized joint laxity, the hyperextended thumb may touch the forearm. Both genu and cubitus recurvatum may also be present [27]. FF with generalized joints laxity is also associated with congenital benign hypotonia, a neurological disease whose pathogenesis of which is still unknown [29].

Furthermore, we still do not know why, in most FFT, the laxity of both capsules and ligaments of the longitudinal arch of the foot disappears and their normal strength is recovered during late childhood and adolescence. However, sex hormones might play a role in this structural change [93].

Severe FFT with diffuse ligamentous laxity is associated with diseases of the connective matrix such as Marfan disease, Ehlers–Danlos syndrome, and trisomy 21 or Down syndrome. The former is caused by the absence of fibrillin, a protein forming the outer layer of the elastic fibers; Ehlers–Danlos syndromes are caused by either a defect of the collagen structure or of its state of aggregation, while joint laxity in Down syndrome is caused by an increased synthesis of collagen type VI [30,31,32]. It may be assumed that the cause of the FF ligament’s laxity will eventually be discovered, as has occurred for the above-mentioned genetic diseases.

Both children and adolescents with high BMI are at risk of developing FF, genu valgum and lower limb joint pain [28]. They have less body control and functional ability and, quite often, poor coordination of movements. Obese children typically lead a sedentary lifestyle. They tend to refuse sports and any form of physical activity. The treatment of this condition is complex, involving mainly pediatricians and psychologists. It is evident that curing only FF plays a marginal role in this complex clinical condition [94].

The pathophysiology of FF is rather complex. Tarsal ROM is increased according to the Huson law [34], as is tibio-tarsal ROM, due to ligament laxity. Consequently, the talus shifts both medially and plantarly, and the malleolar mortise rotates internally to maintain the correct relationship of the tibio-tarsal joint, while the forefoot pronates to guarantee correct loading on the metatarsal heads. At the same time, the calcaneus shifts in valgus at the subtalar joint and, in long-standing cases, the consequent retraction of the triceps’ surae–tendo Achillis unit makes the Silfverskiöld sign positive [12,13,15]. Lower limb rotational deformities are also present in people with FF. In those cases, there is a significant decrease in the strength of hip external rotators, internal rotators, flexors, and extensors, which contributes to poor control of hip position [33].

Pain is the typical symptom of symptomatic FF. In some cases, excessive strain on some muscle-tendon units and capsules and ligaments on the medial side of the tibio-tarsal and tarsal joints, as well as overloading on the subtalar joint, can cause ankle and foot pain. High BMI, high levels of activity, improper footwear, and age-related loss of elasticity in tendons and ligaments may be determinant contributing factors for triggering pain in patients with FF.

Fatigue is the other symptom characterizing symptomatic FF. The current consensus is that the collapsed arch causes extra energy expenditure due to the lack of ground reaction force and consequent muscle imbalance, with some foot activators working more than others, which are inactive during gait [43].

Recent electromyographic and gait analysis studies have shown that foot muscle activity and foot ground contact in FFT are different from normal-arched feet [44,45]. However, different biomechanical behavior does not indicate pathological behavior producing either immediate or delayed symptoms like pain and fatigue, as shown by epidemiological studies [15,16,17,18,19,21]. However, in cases of easy fatiguability, all neuromuscular diseases must be ruled out [52], and the examining orthopedist should perform a careful diagnostic reassessment.

FF diagnosis is based first on a physical examination. In the standing position, the longitudinal arch of the foot is absent, but it reappears either after having elicited the Jack’s test or when the patient walks on tiptoes. In the lying position, with the patient prone and with foot manipulation bringing the hindfoot in supination and the forefoot in pronation, the longitudinal arch easily recovers because all of the foot joints are souple. Only in severe and long-standing FFT is may maneuver be unsuccessful because the alteration of the morphology of the tarsal joints hinders the re-establishment of the normal anatomic articular relationships. All of the other dynamic tests are negative in asymptomatic FF, including endurance tests, such as jumping on one foot at a time, that conversely may become positive in truly symptomatic FF patients.

Standing radiographs of the foot are useful to confirm diagnosis in doubtful cases [22], while dynamic radiographs and weight-bearing CT scans may be useful for both diagnosis and treatment [38,39]. On the contrary, footprints might provide false positivity in young children [12,13]. MRI is indicated either for differential diagnosis with pathologic FF or when abnormalities of the subtalar joint are suspected [40].

Differential diagnosis between FF and pathological flatfoot is easy because the latter is rigid and is not correctable by manipulation, as is FF. The only exception is peroneal spastic FF, which is stiff like pathological flatfoot. In that case, MRI is needed to evaluate the presence of tarsal coalitions that are pathognomonic of pathological flatfoot [12,46].

However, a crucial point is to make a correct differential diagnosis between a truly painful FF and other painful pathological conditions of the foot that may coexist with an asymptomatic FF, thus transforming it into a symptomatic FF. The most frequent pathological conditions needing differential diagnosis are Sever disease (Figure 1), Kohler I disease, accessory tarsal navicular, os trigonum syndrome, plantar fasciitis, growing pains, Freiberg disease, osteochondral lesions of the talus, osteoid osteoma of the foot bones and chronic ankle sprain (Figure 2). In overweight children and adolescents with FF leading a sedentary life, as well as in young people who seek surgery to improve their sport performance, when the referred symptoms do not correspond to the objective clinical signs, the physician should proceed with caution. In both cases, pain perception and treatment expectations may be influenced by psychosocial factors, body weight, family concerns, activity level, cosmetic concerns, and unrealistic expectations regarding surgery. As expert opinion, those clinical observations cannot be included in evidence-based conclusions.

Another important aspect is the esthetic appearance of FF, which mainly affects adolescent girls in whom FF may cause significant anxiety. Moreover, girls often have difficulty finding narrow shoes, and therefore they believe that surgery may solve this problem by modifying their FF into a thin-shaped foot. However, we have not been able to find, in the current literature, any reference for this clinical problem in relation to FF.

Indications for treatment should be carefully evaluated in a condition like FF that can be considered a variation of foot development; in fact, most FFT do not cause pain or functional limitations [12,13]. In symptomatic cases, conservative treatment should be first tried. According to recent systematic reviews and meta-analyses, customized insoles, particularly those made with the 3D-printed technology [55,57,58], either alone or associated with strengthening exercises of both leg and foot muscles [94], may be beneficial. However, a single universally recognized percentage of symptomatic FFT responding to insole treatment at various ages is not available, owing to the heterogeneity of research. In a randomized controlled trial of children with symptomatic FF, evaluated by PODCI and PedsQL questionnaires, Hsieh et al. [95] reported an improvement of both pain and function in 76.2% of cases with customized insoles worn for 12 weeks. On the other hand, Oerlemans et al. [56] carried out a systematic review and meta-analysis of both children and adults with FF treated by insoles, including only randomized controlled trials (RCTs) and prospective studies with a control group. Three out of four RCTs, including only children evaluated with validated scales, showed significant improvements of FF pain and function. However, owing to the heterogeneity of the outcome measures, the authors only considered, for their meta-analysis, three RCTs of adults with FF that used VAS as the outcome measure. The conclusion was that insoles provided a significant pain improvement only in adults.

It is even more difficult to give surgical indications for FF [14]. At first sight, this does not seem to be true, as shown by the numerous studies published in the last 20 years on LIS for the treatment of FF—most of them coming from Europe, North Africa, Asia, and South America (Table 1). The high number of symptomatic cases undergoing surgery every month, ranging from 2 to 13 cases with the risk of including misdiagnosed cases, seems to be in contrast with the low number of symptomatic cases reported by epidemiological studies [15,16,17,18,19,20,21]. However, we must consider that epidemiological studies reflect general populations, while surgical series represent selected surgical cohorts. Without considering this selection mechanism, the discrepancy between the data obtained from FF epidemiology and LIS can appear overstated. Another cause for concern is age at surgery: although the average age of the surgical cohorts ranges from 9.5 to 13.7 years, children ranging in age from 5 to 8 years are also included in almost all the surgical series (Table 1). However, the longitudinal arch of the foot is not completely formed before 9–10 years of age, which should also represent the minimum age for LIS. We are also concerned because, in some studies, surgery was not only indicated by symptomatic FF. Roth et al. (Table 1) reported on 94 FFT with and without pain evaluated by altered radiographic angles that normalized after SA in almost 97% of cases. De Pellegrin et al. (Table 1) reported on 732 FFT. Inclusion criteria for SA were not only pain and short Achilles tendon, but also Staheli arch index > 1 or from two to three altered radiographic angles. Franz et al. (Table 1) indicated surgery in 82 FFT by abnormal pedobarography that normalized following SA.

Most of the studies on LIS have a low level of evidence (IV–V). Furthermore, they vary by study design, length of follow-up, outcome measures, and complication rate, and no study reports the ratio between the operated FFT and the overall number of FFT observed in the clinic. In addition, screws used for exosinotarsal arthroereisis vary across the studies for both size and design (either cortical, or cancellous or cannulated), as well as for composition (stainless steel, titanium, bioabsorbable). The same is true for the implants used for endosinotarsal arthroereisis, which also vary in both design and composition (titanium, bioabsorbable polylactic acid, mixed titanium and polyethylene). The only factor that links all of these studies is the surgical outcome, which is either excellent or good in a very high percentage of cases, regardless of the results evaluation method (Table 1). For all of these reasons, we believe that no definite conclusion can be drawn from these studies.

Finally, LIS is not immune to complications, which may be either severe, requiring repeated surgery, or mild, requiring only conservative treatment. They vary according to the specific technique of SA—exosinotarsal or exosinotarsal—as well as to the material of the implant, either metallic or bioabsorbable. The percentage of the most frequent complications is reported in Table 1, although their direct comparison is limited by variability in definition, used implant and length of follow-up across studies. Higher quality studies are needed for a more precise definition of the risk of complication in FF LIS.

Comparative studies between exosinotarsal and endosinotarsal SA do not show significant differences in terms of outcomes, but rather in terms of complications that are different (Table 1). From an economic standpoint, exosinotarsal technique with a metallic screw has the lowest cost [81].

**Figure 4:**
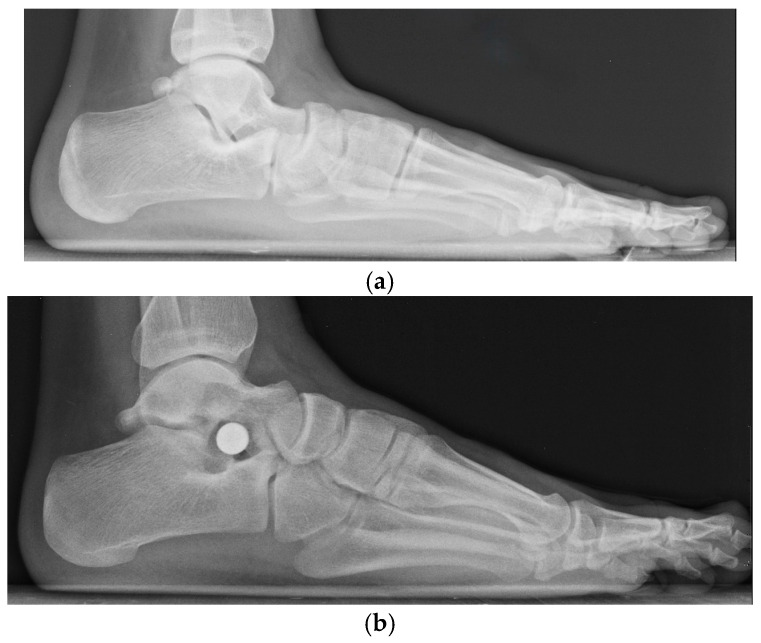
(**a**) Standing lateral radiograph of the left symptomatic flatfoot of a 12-year-old girl. Meary’s and pitch angles measured, respectively, −16° and 6°. (**b**) The same foot four months after the application of an endosinotarsal implant. The foot was asymptomatic, and Meary’s and pitch angles measured, respectively, 0° and 15°.

Our experience with FF surgery is in line with the North American pediatric orthopedic surgeons who only recently adopted LIS [89]. We gave indication for surgery to only 26 FFT during a 10-year period, and the age of the patients ranged from 11 to 14 years [90], while in North America, Sullivan at al. operated with exosinotarsal screw SA on only 37 FFT in 5 years and the age of their patients ranged from 9 to 13 years [89]. We also believe that LIS has no universal indication, and therefore severe FF long-standing cases in adolescents might need other reconstructive surgical techniques when both Jack’s test and tiptoe-walking, as well as dynamic diagnostic imaging, do not show restoration of the longitudinal arch of the foot [13,90]. Nonetheless, SA with the proper indication provides very good results by restoration of the medial longitudinal arch with the least surgical invasiveness, mainly in cases of marked ligament laxity [3,61] (Figure 4). In cases of tight Achilles tendon with prevalent calcaneus valgus deformity, Achilles tendon lengthening alone may be indicated if conservative treatment by stretching exercises fails [12].

The most important limitation of our narrative review is the low level of clinical relevance of many of the quoted articles dealing with LIS. In fact, most of them reach a level of evidence of IV–V, while most cross-sectional epidemiological studies, as well as many pathogenetic, pathological, electromyographic, clinical, and conservative treatment studies, often reach a higher level of evidence. Therefore, most of the studies on SA are mainly biased by different cohort sizes, different result evaluation methods, different lengths of follow-up, and lack of a control group to compare surgical outcomes. Readers are then advised to focus more on the clinical and radiographic outcomes rather than on statistical comparison and level of evidence of those studies. Future prospective randomized studies are needed to compare the advantages and disadvantages of FF surgical treatment with non-surgical approaches by long-term follow-up studies.

## 5. Conclusions

In summary, the definition of FF as a crippling deformity is supported by mixed evidence. Most FFT are asymptomatic during childhood, adolescence, and adulthood, and they do not need any treatment. In symptomatic cases, after careful diagnostic screening to avoid misdiagnosis with other coexisting diseases, conservative treatment is first indicated. In case of failure, surgery must be performed in residual cases by choosing the most appropriate technique according to the clinical-radiographic features of the individual FF case. SA may be considered in carefully selected children and adolescents because it is technically less demanding and has been associated with satisfactory outcomes in several series.

**Figure 5:**
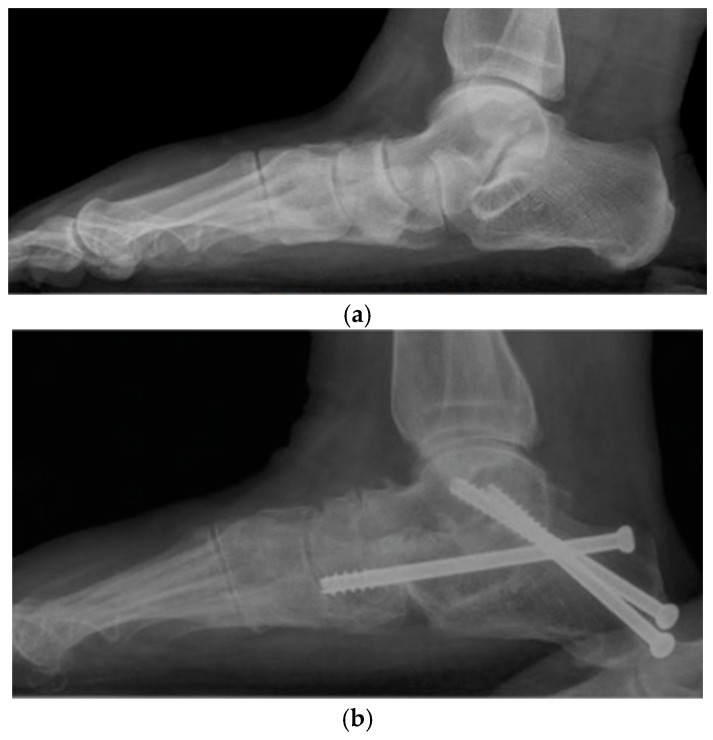
(**a**) Standing lateral radiograph of the right foot of a 48-year-old patient whose flatfoot recently became painful following a marked increase in body weight. (**b**) A triple arthrodesis was performed, with partial radiographic correction of the flatfoot and disappearance of pain.

**Figure 6:**
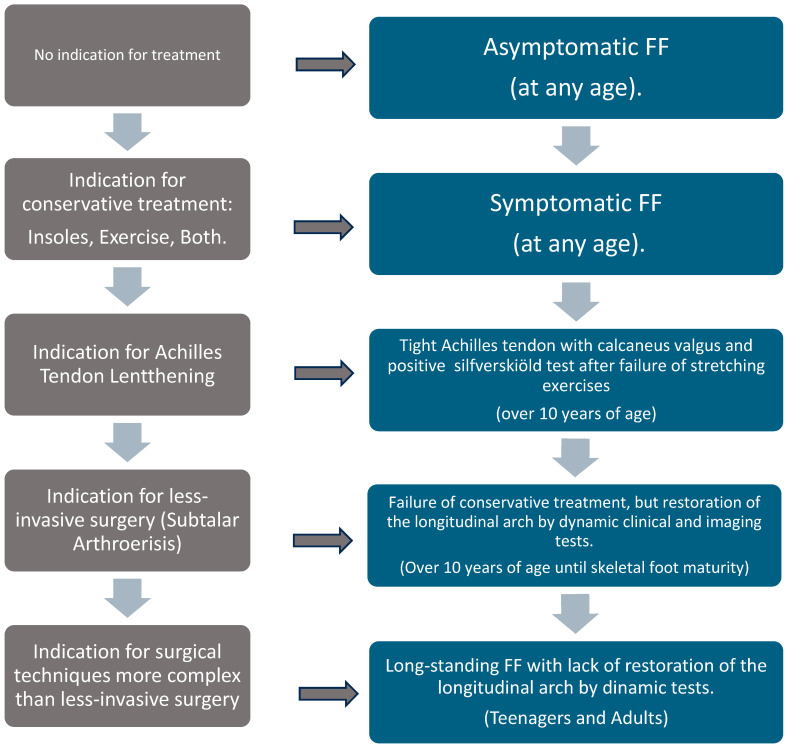
Flowchart of flexible flatfoot (FF) management.

However, indication should be appropriately given to avoid overtreatment. In addition, parents must be reassured of the benignity of FF and not be burdened with the responsibility of preventing its unfavorable evolution into adulthood, firstly because such an evolution has so far only been supposed, and secondly because FFT that become symptomatic in adulthood may also be treated surgically with satisfactory results [91] (Figure 5). Based on the conclusions of this review, we report the indications for FF management in Figure 6.

## Figures and Tables

**Table 1:** Operated flexible flatfeet (FFT) published from 2007 to 2025. Exosinotarsal arthroereisis: EX; endosinotarsal arthroereisis: EN. In studies in which several evaluation methods of the outcomes were used, the most reproducible methods were selected. Standing foot radiographic angles: SFRA.

Study (Author, Year, Journal) and Surgical Technique	Patients’ Age in Years (y)	Symptoms: Definition and Indication	Attempted Conservative Treatment	Length of Follow-Up in Years (y)	Outcomes Evaluation Method	Outcomes	Complications
Roth S. et al., 2007 (Foot Ankle Int.) [62]; EX	Range (8–14 y); 94 FFT	Symptomatic FFT, asymptomatic FFT with altered radiographs	Yes, strengthening exercises and orthoses	Mean: 5 y	Authors’ own scale (1 to 10 points)	91% excellent/good; 9% poor.	23.57%
Fernandez de Retana P. et al., 2010 (Foot Ankle Clin.) [64]; EN	Range (7–14 y); 97 FFT	Symptomatic FFT	Not mentioned	Mean: 4.5 y	AOFAS scale; SFRA	Significant improvement in both AOFAS scale and SFRA.	29.9%
Pavone V. et al., 2013 (J Foot Ankle Surg.) [66]; EX	Range (7–14 y); 410 FFT	Symptomatic FFT	Yes, orthoses	Mean: 7.3 y	Visual Analog Scale (VAS); SFRA	VAS: 81.71% excellent, 15.12% good, 3.17% poor; Significant SFRA improvement.	12.86%
De Pellegrin M. et al., 2014 (J Child Orthop) [67]; EX	Range (5–17.9 y); 732 FFT	Symptomatic FFT; Staheli arch index >1; 2 on 3 pathologic SFRA	Yes (not specified)	Mean: 3.1 y	Authors’ own clinical evaluation method; SFRA	93.7% good, 6.3% poor. Significant SFRA improvement.	3.4%
Calvo Calvo S. et al., 2015 (Rev. Esp. Cir. Orthop.) [69]; EX	Range (7.11–14.8 y); 103 FFT	Symptomatic FFT	Yes, orthoses	Mean: 15.66 y	LICKERT questionnaire in 54% of patients; SFRA in 34% of cases	93% very satisfied and satisfied, 7% less satisfied and unsatisfied. SFRA improvement, significant for only one angle.	10%
Giannini S. et al., 2017 (J Foot Ankle Surg.) [71]; EX	Range (8–14 y); 88 FFT	Symptomatic FFT	Not mentioned	Mean: 4.6 y	Authors’ own clinical evaluation method; SFRA	75% excellent, 20.5% good, 4.5% poor; significant SFRA improvement.	2.2%
Arbab D.F. et al., 2018 (Z. Orthop. Unfall.) [72]; EX	Range: (9–14 y); 73 FFT	Symptomatic FFT; failed conservative treatment	Yes (not specified)	Mean: 2.5 y	Authors’ own questionnaire; SFRA	95% very satisfied and satisfied; SFRA significant improvement, but one angle.	14.1%
Memeo A. et al., 2018 (J Foot Ankle Surg.) [74]; Comparative study Group A: EX versus Group B: EN	Range (8–16 y); 402 FFT	Symptomatic FFT with Viladot’s grade 3 and 4 footprints	Not mentioned	Median: 10.8 y	Authors’ own clinical evaluation method; SFRA	Clinical improvement in all patients. SFRA normalization in almost all cases. No significant difference between the two procedures.	14.8% in Group A and 12.5% in Group B
Pavone V. et al., 2018 (J. Child. Orthop.) [73] EX	Range (9–15 y); 136 FFT	Symptomatic FFT in recreation sport practitioners	Yes, orthoses	Mean: 4.8 y	AOFAS Scale, OxAFQ-C, FADI Sport questionnaires; SFRA	AOFAS, OxAFQ-C and FADI Sport significant improvement; SFRA normalization.	5.9%
Kubo H. et al., 2019 (J Orthop Sci) [77] EX	Range (5–15 y); 149 FFT	Symptomatic FFT	Not mentioned	Mean: 2.9 y	Three standing biplane radiographs	Significant improvement of radiographic parameters in patients within age range 9–12 y.	Not mentioned
Elmarghany M. et al., 2020 (J Orthop) [76] EX	Range (7–15 y); 84 FFT	Symptomatic FFT	Not mentioned	Mean: 2.4 y	AOFAS scale; SFRA	Both AOFAS scale and SFRA significant improvement.	0.07%:
Franz A. et al., 2021 (Foot Ankle Surg) [79] EX	Mean: 11.3 ± 1.4 y; 78 FFT	Abnormal pre-op pedobarography; to investigate pedobarographic changes after EX	Not mentioned	Mean: 8 months	Pedobarography	Pedobarography normalization after EX.	Not mentioned
Mazzotti A. et al., 2021 [80] (Int. Orthop.) EN	Range (9.2–14.9 y); 64 FFT	Symptomatic FFT	Not mentioned	Mean: 15 ± 2.7 y	AOFAS and SF-12 scales; SFRA	AOFAS: 94.1% excellent/good, 5.9% fair; SF-12: high average value (44.7 ± 3.2); SFRA significant improvement.	8.2%
Vogt B. et al., 2021 (CHILDREN) [78] Comparative study: EN with two different implants versus EX with metallic screw	Range (5–16 y); 113 FFT	Symptomatic FFT	Yes (not specified)	Mean: 2.4 y	Foot Function Index (FFI), pedobarography, SFRA	FFI, pedobarography and SFRA significant improvement. No significant difference among the three procedures.	29% EN with Kalix implant; 7% EN with bioabsorbable implant; 11% EX with metallic screw
Zahid et al., 2021 (J. Appl. Sci.) [81] Comparative study: EX versus EN	Range: (5–15 y); 60 FFT	Symptomatic FFT	Yes (not specified)	Mean: 1.6 y	AOFAS scale; SFRA	Both AOFAS scale and SFRA significant improvement. No significant difference between the two procedures.	13.4% EX; 6.7% EN
Ghaznavi A. et al., 2022 (Med. J. Islam. Rep. Iran) [82] EX	Range: (5–15 y); 57 FFT	Symptomatic FFT	Not mentioned	Minimum 1 y	Authors’ own clinical evaluation method; SFRA	96.5% pain relief; SFRA significant improvement.	3.5%
Di Bello D. et al., 2023 (PLoS One) [83] EX	(Range: 8–15 y); 500 FFT	Clinical results, quality of life	Not mentioned	Mean: 3.18 ± 0.98 y	OxAFQ-C; PedsQL™	OxAFQ-C significant improvement; PedsQLTM medium-high score.	Not mentioned
Alexeevich K.G. et al., 2025 (Int. J. Res. Ortho.) [86] EX	(Range: 6–14 y); 130 FFT	Symptomatic FFT; to evaluate loss of correction after screw removal	Yes (not specified)	Mean: 3 years after screw removal	Pedobarography; SFRA	No significant pedobarographic and radiographic loss of correction.	Not mentioned
Manuri V. et al., 2025 (J. Musculoskel. Surg. Res.) [84] EN	Range: (8–19 y); 92 FFT	Symptomatic FFT	Not mentioned	Mean: 3.8 y.	AOFAS scale and FADI	Both AOFAS scale and FADI significant improvement.	Not mentioned
Silva et al., 2025 (J. Exp. Orthop.) [85] EX	Mean: 11.7 ± 1.3 y; 644 FFT	Symptomatic FFT	Not mentioned	Mean: 5.6 ± 1.18 y	Authors’ own questionnaire; Tegner Activity Scale	94% successful outcome, 6% pain persistence; significant improvement in the Tegner Activity Scale with recovery of sports activity.	4.8%

## Data Availability

Data is contained within the article.

## Abbreviations

The following abbreviations are used in this manuscript:

FF	Flexible Flatfoot
FFT	Flexible Flatfeet
LIS	Less-Invasive Surgery
SA	Subtalar Arthroereisis
